# Rupture post traumatique d'un kyste hydatique du foie dans le peritoine

**DOI:** 10.11604/pamj.2016.23.124.9053

**Published:** 2016-03-24

**Authors:** Hedfi Mohamed, Charfi Mehdi

**Affiliations:** 1 Service de Chirurgie Generale, Hopital des Fsi, La Marsa, Tunisie; 2 Service de Radiologie, Hopital des Fsi, La Marsa, Tunisie

**Keywords:** Hydatid cyst, peritonitis, scanner, Hydatid cyst, peritonitis, scanner

## Image en medicine

L'hydatidose péritonéale est l'une des complications les plus graves de la maladie hydatique. Elle est favorisée par le siège du kyste, la taille, et la pression intra kystique élevée. La rupture traumatique est le plus souvent iatrogène lors d'une intervention chirurgicale pour Kyste Hydatique du Foie (KHF), nous rapportons une nouvelle observation d'un patient âgé de 26 hospitalisé pour péritonite aigue en rapport avec une rupture d'un Kyste hydatique du foie dans le péritoine.

**Figure 1:**
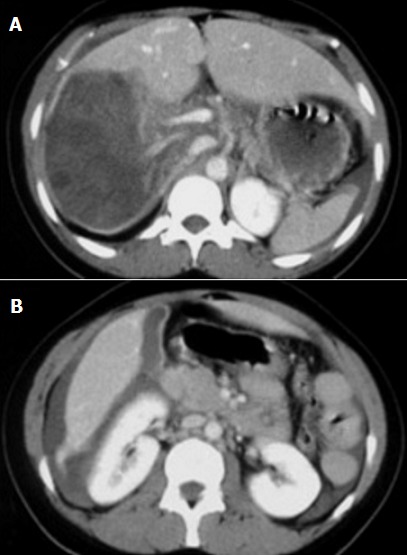
A) TDM du foie avec PC, rupture post-traumatique intrapéritonéale d'un KH du foie, large solution de continuité postéro-interne du KH avec épanchement périhépatique; B) épanchement perihépatique

